# A descriptive study of colorectal function in adults with Prader-Willi Syndrome: high prevalence of constipation

**DOI:** 10.1186/1471-230X-14-63

**Published:** 2014-04-04

**Authors:** Louise Kuhlmann, Iben Moeller Joensson, Jens Broendum Froekjaer, Klaus Krogh, Stense Farholt

**Affiliations:** 1 Department of Pediatrics, Centre for Rare Diseases, Aarhus University Hospital, Aarhus, Denmark; 2 Department of Hepatology and Gastroenterology, Neurogastroenterology Unit, Aarhus University Hospital, Aarhus, Denmark; 3 Department of Pediatrics, Aarhus University Hospital, Aarhus, Denmark; 4 Mech-Sense, Department of Radiology, Aalborg University Hospital, Aalborg, Denmark

**Keywords:** Prader-Willi Syndrome, Developmental disabilities, Gastrointestinal transit, Constipation, Rectum, Colon, Ultrasonography

## Abstract

**Background:**

Some patients with Prader-Willi Syndrome (PWS) have symptoms of constipation, but bowel function in PWS has never been systematically evaluated. The aim of the present study was to describe colorectal function in PWS by means of validated techniques.

**Methods:**

Twenty-one patients with PWS (14 women, age 17–47 (median = 32)) were evaluated with the Rome III constipation criteria, stool diary, digital rectal examination, rectal diameter assessed from transabdominal ultrasound, and total gastrointestinal transit time (GITT) determined with radio-opaque markers. Results were compared with those of healthy controls.

**Results:**

Among PWS patients able to provide information for Rome III criteria, 8/20 (40%) fulfilled the criteria for constipation. Most commonly reported symptoms were a feeling of obstructed defecation (8/19, 42%), <3 defecations per week (8/17, 47%), straining during defecation (7/19, 37%) and lumpy or hard stools (6/19, 32%). Rectal diameter did not differ between PWS (median 3.56 centimeters, range 2.24–5.36) and healthy controls (median 3.42 centimeters, range 2.67–4.72) (p = 0.96), but more PWS patients (13/20; 65%) than healthy controls (3/25; 12%) (p < 0.001) had fecal mass in the rectum. Median GITT was 2.0 days (range 0.5–4.4) in PWS versus 1.6 (range 0.7–2.5) in the control group (p = 0.26). However, GITT was >3 days in 5/21 (24%) of PWS and none of the controls (p = 0.047).

**Conclusion:**

Constipation is very common in PWS. Patients with PWS have an increased prevalence of prolonged GITT and palpable stools in the rectum at digital rectal examination.

## Background

Prader-Willi Syndrome (PWS) is a multisystemic genetic disease which was first described in 1956 [1]. The incidence of PWS is 1:15.000–30.000 newborns [2]. The syndrome is characterized by muscular hypotonia, feeding difficulties and failure to thrive in the early childhood [3]. Further characteristics are developmental delay, short stature and hypogonadism [2]. During childhood a certain demeanor characterized by a food-seeking behavior and hyperphagia develops. Patients may also develop compulsive-like behavior with skin and rectal picking. In severe cases the latter can lead to rectal prolapse and fecal incontinence [3].

Gastrointestinal function in PWS is sparsely investigated. Three studies have found either normal [4,5] or slow gastric emptying in PWS [6]. Also, serious events with gastric rupture and necrosis and other gastrointestinal problems have been reported [7-9]. Other studies have found an increased prevalence of constipation in mentally disabled patients [10-12]. In spite of this, colorectal function, including constipation, has never been systematically evaluated in PWS.

Constipation is normally a symptom based diagnosis. Since 2006, the Rome III criteria have gained general acceptance for defining constipation in a clinical setting [11]. Oral intake of radio-opaque markers followed by one or more abdominal x-rays is a simple and commonly used method for assessment of total gastrointestinal transit time (GITT) [13]. As transit time through the colorectum is much longer than gastric emptying or small intestinal transit time, GITT mainly reflects colorectal transit time and both terms are often used. Some patients have normal or almost normal GITT but still suffer from severe symptoms of constipation. This is often caused by disordered rectal evacuation and estimation of rectal diameter by transabdominal ultrasonography (US) has been introduced for non-invasive and risk free evaluation of rectal impaction in children [14-17].

Based on clinical experience, we hypothesized that constipation is common in PWS. Accordingly, the aim of the present study was to describe symptoms of constipation in a group of adult patient with PWS and to compare commonly used parameters of colorectal function in patients with PWS and healthy controls.

## Methods

The study was performed in accordance with the Helsinki II declaration and approved by the Central Denmark Region Committees on Health Research Ethics (registration number 20110043) and the Danish Data Protection Agency.

Among 28 PWS patients consecutively seen at the outpatient clinic at Centre for Rare Diseases, Aarhus University Hospital, 21 (14 women, age 17-47 years (median 32)) with a BMI of 23.6 (18.1–43.0) were included in the present study. Eight patients were obese as defined by a BMI > 25. All patients, their GP and their guardian gave written consent to participate in the study. Seven were excluded because of previous major abdominal surgery (n = 1), treatment with medications affecting gastrointestinal motility (n = 3), failed genetic diagnosis (n = 1), or inability to follow the study protocol (n = 2). In 19 patients PWS was caused by a deletion and in two uniparental disomy. Patients were evaluated with the Rome III constipation criteria and they were helped to complete a two-week stool diary. A clinical examination including digital rectal examination and US were performed in each subject. Finally, GITT was determined with radio-opaque markers as described below.

For comparison of rectal diameter determined with US, we recruited 30 healthy volunteers (16 women, aged 20-67 years (median = 26)) with a BMI of 23.1 (17.7–30.0) without previous major gastrointestinal surgery and without medication or present disease affecting gastrointestinal function. None of the healthy controls were constipated according to the Rome III constipation criteria.

The control subjects were scanned by 3 doctors, including one experienced radiologist, and no significant inter- or intra-observer variability were found.

For ethical reasons and to reduce exposure of healthy subjects to irradiation, GITT in PWS patients were compared to existing normative data from our unit (16 healthy adults, 12 men, aged 23-60 years (median 31)).

### Rome III constipation criteria

The Rome III criteria cover various symptoms of constipation [13]. The patients completed a questionnaire in plain language covering Rome III criteria together with a parent or a care assistant from their group homes. The helpers could thereby clarify the meaning of the questions, if the patients had trouble understanding them.

### Stool diary

For a period of 14 days the patients were helped to register every defecation, passing of water and episode of fecal and urinary incontinence by marking in a date and time table divided into columns headed by correlating drawings of a toilet, stools, urine and underpants, respectively. Data of stool consistency was not obtained in purpose of minimizing the risk of provoking their food-seeking behavior towards their stools.

### Clinical examination

The clinical examination included perianal inspection and digital rectal examination. We examined the position of anus, presence of perianal feces, reddening, eczema, fissures, haemorrhoids, perianal sensibility, anal sphincter tone, voluntarily contraction/relaxation of the anal sphincter and the amount and consistency of feces in the rectum.

### Gastrointestinal transit time

Assessment of GITT was performed according to Abrahamsson et al. etc. [13,15,17,18]. Subjects took six gelatine capsules each containing 10 radio-opaque markers each morning for six consecutive days. On the seventh day an X-ray of the abdomen was taken and the number of radio markers was counted. From this the GITT in days was calculated as:

$$\mathrm{GITTd}=\frac{M+\left(\mathrm{fxD}\right)}{D}$$

where f is the fraction of the daily dose chosen for estimation of GITT. We have chosen to set f to 0.5 as in earlier studies [17,19]. D is the number of radio-opaque markers per day, in our case 10, and M is the amount of remaining markers on the X-ray.

The segmental colonic transit time (SCTT) of the right (caecum, ascending and transverse colon) and the left colorectum (descending colon and rectosigmoid) were calculated as [20,21]:

$$\mathrm{SCTT}=\frac{M}{10}$$

### Ultrasonic measurement of rectal diameter

With subjects in the supine position the scanner (MINDRAY M5 Portable Ultrasound Scanner, Mindray, Shenzhen, China) with an abdominal curved 5 MHz probe was placed a few centimeters above the symphysis and the probe was angled 20 degrees towards os sacrum. Confirming that the bladder was not empty and, thereby, having acceptable conditions for imaging, the rectal diameter was measured as the transverse distance (right to left) of the outer rectal contour and by carefully identifying its widest part. The interface between the hypoechoic muscular wall and the hyperechoic perirectal fat was used. This was done three times and the mean value was calculated. After US perianal inspection and digital rectal examination were performed and it was noted whether palpable stools was present.

### Statistical analysis

The Mann-Whitney U-test was used for comparison of groups. The proportion of subjects with GITT > 3 day was compared by the Fishers exact test. Correlation was tested by the Pearson test of linear correlation. P < 0.05 was considered statistically significant. The statistical calculations were performed with R 2.14.1 software (R Foundation, Vienna, Austria).

## Results

Twenty of 21 PWS patients provided reliable information about the Rome III criteria and among these, eight (40%) fulfilled the definition of constipation. Even though the group of PWS patients had a median number of bowel movements per day of 1.6 (range 1–3.8), specific constipation related symptoms were frequent (Table 1). In total, only two episodes of urinary incontinence and one episode of fecal incontinence were reported during a two-week period.

**Table 1:** The Rome III constipation criteria

**Criteria (at least 25 % of defecations)**	**Frequency of symptom**
Straining during defecation	7/19 (37%)
Lumpy or hard stools	6/19 (32%)
Sensation of incomplete evacuation	3/20 (15%)
Sensation of anorectal obstruction/blockage	8/19 (42%)
Manual manoeuvres to facilitate defecation	0/20 (0%)
Fewer than 3 defecations per week	8/17 (47%)
Loose stools are rarely present without laxatives	3/20 (15%)
Insufficient criteria for IBS (irritable bowel syndrome)	20/20 (100%)

**Figure 1:**
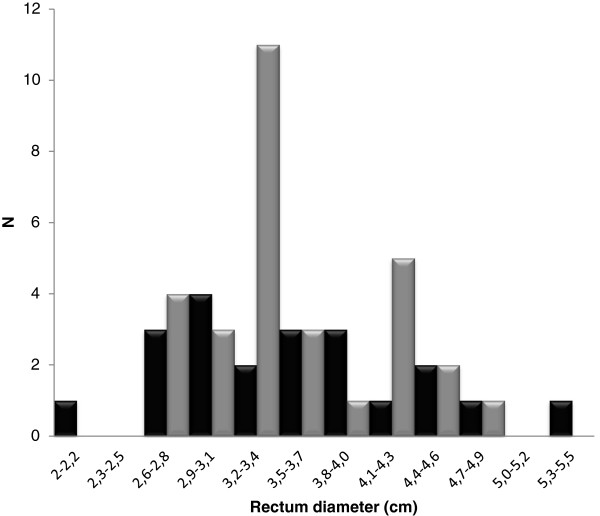
**Rectal diameters in adults with Prader**-**Willi Syndrome (black) and healthy adult controls (grey).**

Rectal diameter did not differ between PWS (median 3.56 centimeters, range 2.24–5.36) and healthy volunteers (median 3.42 centimeters, range 2.67-4.72) (p = 0.96) (Figure 1). However, more PWS patients (13/20; 65%) than healthy volunteers (3/30; 10%) (p < 0.001) had fecal mass in the rectum at digital examination. No fissures, eczema, haemorrhoides or rectal bleeding were observed neither in the PWS nor in the control group. Likewise the perianal sensibility and anal sphincter tone were normal in both groups.

**Figure 2:**
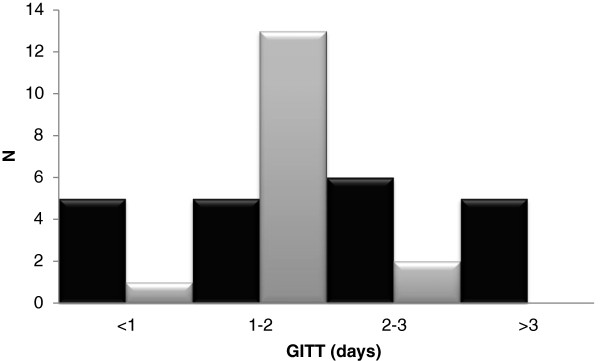
**Total gastrointestinal transit times in days in adults with Prader**-**Willi Syndrome (black) and healthy adult controls (grey).**

Median GITT was 2.0 days (range 0.5–4.4) in PWS versus 1.6 (range 0.7–2.5) in the control group (p = 0.26). However, GITT was >3 days in 5/21 (24%) of PWS and none of the controls (p = 0.047) (Figure 2). Median transit time of the right colon was 0.6 days (range 0-2.2) compared to 0.4 days (range 0–1) in healthy volunteers (p = 0.32). Median transit time of the left colorectum was 0.9 days (range 0-2.6) in PWS patients and 0.6 days range (0.2–1.2) in controls (p = 0.48).

There was a trend towards a correlation between Rome III evaluation and presence of feces in the rectum (p = 0.09, r = 0.38) but not between Rome III and GITT (p = 0.39, r = 0.20). Likewise, there was no correlation between rectal diameter and presence of feces in the rectum (p = 0.83, r = -0.3). There was no difference between male and female PWS patients in terms of GITT (p = 0.75), rectal diameter (p = 0.58) and number of Rome III criteria met (p = 0.74). Furthermore, we did not find any correlation 213 between BMI and signs of constipation (palpable stools 214 in the rectum (p = 0.47) and GITT (p = 0.34)).

## Discussion

The main finding of the present study was that adult patients with PWS had a surprisingly high prevalence of constipation related symptoms compared to that reported in the general population [21]. Congruently, significantly more PWS patients than healthy controls had prolonged GITT and stools in the rectum. The study design was descriptive and does not allow any conclusions about the pathophysiology of constipation in PWS.

Patients with PWS have abnormal eating habits. However, all patients but two in the present study lived at institutions with daily routines specially adapted to PWS, including a fixed healthy diet. Therefore, diet cannot explain our results. Recollection bias may be a problem, especially in persons with intellectual deficits, and stool diaries may have been incomplete in spite of help from caretakers. However, the objective methods used supported the symptom based data.

Symptoms of constipation are common and 11% of the general population fulfills the Rome III criteria [21].This is, however, still much less than the 40% observed in the present study. Our finding is in line with a self reported life-time prevalence of constipation of 37% described in a recent paper [22]. Objectified with radiographic colonic transit time, the prevalence of slow colonic transit is 2-4% in the general population, when slow transit constipation is defined as a GITT above 3 days [23,24]. This is in contrast to the 24% prevalence observed among PWS patients in the present study.

We can only speculate about the pathophysiology behind constipation in PWS. The presence of stools in the rectum indicates a rectal evacuation dysfunction. This was, however, not supported by the SCTT, where prolonged transit of the left colon should be expected in case of a severe evacuation disorder. Normal defecation is preceded by colonic mass movement and sensation of stool in the rectum. Abnormal rectal sensation would be coherent with the typical PWS characteristic of abnormal conception of physiological signals from the body [2,3,9]. Patients with PWS usually have reduced tone of striated muscles. Reduced tone of the smooth muscle cells within the colorectum may lead to constipation including incomplete rectal evacuation. Further studies including evaluation of rectal sensation and biomechanics are needed to clarify this.

In various patient groups, including irritable bowel syndrome and connective tissue disorders, constipation is part of general gastrointestinal dysmotility [18,19]. It is unknown whether this is the case in PWS too. Furthermore it is debatable whether gastric emptying is prolonged [6] or not [4,5] and small intestinal transit has not been evaluated. Gold standard for evaluation of small intestinal transit is scintigraphy which is not widely available and exposes the subject to irradiation. New and minor invasive methods suitable for studying small intestinal transit in PWS include the Wireless Motility Capsule [25] and the magnet based Motility Tracking System [26].

Ghrelin is an orexigenic hormone and the amount of ghrelin in gastric tissue [27] as well as in plasma [28] is higher than normal in PWS. Ghrelin has a prokinitic effect stimulating gastrointestinal motility. Thus abnormal gastrointestinal function in PWS must be caused by another mechanism [4].

Assessment of rectal diameter by a transabdominal US is sensitive for diagnosing rectal impaction in children [14]. We established a normal adult material and we confirmed that intra- and inter-observer validity were good (unpublished data) before investigating the PWS patients. In both groups there was no correlation between rectal diameter and presence of stools in the rectum. Further studies are, however, needed before introducing US in clinical evaluation of constipation in adults.

Gastrointestinal symptoms including constipation are more common in obese children compared to normal-weight children [29,30]. However, a correlation between obesity expressed as high BMI and constipation in adults is more controversial and most studies cannot confirm this association [23,31].

## Conclusion

We found that 40% of adult PWS patients have constipation according to the Rome III criteria which is considerably more than reported in the general population. Also, PWS patients have an increased prevalence of palpable stools in the rectum and a prolonged GITT. Given that PWS patients often lack the ability to interpret body signals as well as expressing symptoms it is necessary for parents, caretakers and clinicians to pay special attention to bowel related symptoms. Based on our findings, a thorough clinical history combined with a physical examination including rectal digital examination is sufficient to diagnose constipation in PWS. In addition, GITT can be considered. Abdominal US does not provide any additional information.

## Competing interests

The authors declare that they have no competing interest.

## Authors’ contribution

All authors have participated in designing the study, practical evaluation of patients or healthy volunteers and preparation of the manuscript. LKF was main coordinator in the project and prepared the first draft for the manuscript. She took part in all aspects of the study. SF initiated the study and was main supervisor. She also took part in the recruitment of patients, informing the nearest relatives and most of the examinations. KK participated in devising the clinical design of the study. He was also involved in measuring the GITT. IMJ was involved in conceiving the study, the induction training for using the ultrasonic scanner and in scanning the control group. JFB was involved in scanning the control group and the statistical calculations. All authors read and approved the final manuscript.

## Pre-publication history

The pre-publication history for this paper can be accessed here:

http://www.biomedcentral.com/1471-230X/14/63/prepub
